# Thyroid Dysfunction and Chronic Kidney Disease: A Study Among the Northeastern Population of India

**DOI:** 10.7759/cureus.38700

**Published:** 2023-05-08

**Authors:** Md Kashif, Md S Hussain, Mudassir Anis, Papu K Shah

**Affiliations:** 1 Biochemistry, Radha Devi Jageshwari Memorial Medical College & Hospital, Muzaffarpur, IND; 2 Physiology, Mahatma Gandhi Medical College & Hospital, Jaipur, IND; 3 Physiology, Rohilkhand Medical College and Hospital, Bareilly, IND

**Keywords:** chronic kidney disease, thyroid dysfunction, hypothyroidism, thyroid hormones, hemodialysis, egfr, ckd

## Abstract

Introduction: Chronic kidney disease (CKD) is a multifaceted non-communicable disease characterized by a progressive decline in kidney function ultimately requiring renal replacement therapy (RRT) in most patients. Due to the high cost and availability of a limited number of donors, the majority of patients depend on dialysis and conservative management. Thyroid hormones are indispensable for the growth, development, and homeostasis of our body. The kidney plays an important role in the metabolism, degradation, and excretion of thyroid hormones. Various studies have revealed significant dysfunction in thyroid hormone status in CKD patients, but the results are inconsistent.

Aims: To evaluate and compare the thyroid hormone status in CKD patients with healthy controls along with a comparison of thyroid hormones in CKD patients on regular hemodialysis with those on conservative management.

Materials and methods: The present cross-sectional study involved 100 subjects of both sexes between 40 and 70 years of age, out of which 50 were patients of stage 5 CKD with no previous history of any thyroid disorders, while 50 apparently healthy subjects served as control. Of the CKD patients, 52% were on regular hemodialysis while 48% were receiving conservative care. The participants were investigated for various biochemical parameters like blood urea, serum creatinine, total triiodothyronine (TT3), total thyroxine (TT4), and thyroid stimulating hormone (TSH). The estimated glomerular filtration rate (eGFR) was calculated using a modification of diet in renal disease (MDRD) 4 variable formula. The thyroid profiles were also compared between patients of CKD receiving conservative management and those on maintenance hemodialysis.

Results: Of the total sample, 35 (70%) were male and 15 (30%) were female in each of the case and control groups. The mean age of CKD patients and the control group was 55.32 ± 9.62 years and 54.48 ± 9.63 years, respectively. TT3 was reduced in all 50 CKD patients. TT4 was normal in 31 (62%), reduced in 18 (36%), and high in one (2%) case. TSH was high in 38 (76%) cases, while reduced in one (2%) and normal in 11 (22%) cases. The mean blood level of TT3 and TT4 showed a statistically significant reduction (P < 0.0001 for each), while the TSH level showed a significant increase with a p-value of 0.0002 in CKD patients compared to controls. The mean blood urea and serum creatinine levels were statistically increased in cases than in controls (P < 0.0001). The thyroid hormone status revealed a significant difference between CKD patients on maintenance hemodialysis compared to those on conservative care with a p-value of 0.0005 for TT3, 0.0006 for TT4, and 0.0055 for TSH.

Conclusion: Patients with CKD were at risk of thyroid hypofunction irrespective of their mode of treatment. This study highlights the clinically relevant interactions between renal and thyroid function, which may be helpful to clinicians for optimal diagnosis and management of CKD patients.

## Introduction

India has experienced an epidemiological transition with a decline in communicable diseases and a growing burden of chronic diseases [1]. Unhealthy lifestyles, increasing urbanization, and societal and environmental changes have been implicated in the rapid rise in chronic diseases like cardiovascular disease, diabetes mellitus, cancer, and chronic respiratory diseases [1]. Notably, chronic kidney disease (CKD) was not included in that category.

CKD is characterized by a sustained estimated glomerular filtration rate (eGFR) of <60 ml per minute per 1.73 m^2^ of body-surface area for more than three months irrespective of the underlying cause while glomerular filtration rate (GFR) <15 ml/min/1.73 m^2^ is considered as end-stage renal disease (ESRD) [2]. CKD is classified into five stages ranging from mild dysfunction to complete failure [3]. CKD has been identified as a leading cause of morbidity and mortality worldwide [4]. In developing countries like India, between 2001-2003 and 2010-2013, there was a rise of 38% in mortality attributed to renal failure [5]. The epidemiology of CKD in India has been found to be different from the West; the patients were relatively younger by five to 20 years [6]. The probable causes include malnutrition during pregnancy, environmental factors, genetic factors, or delayed medical attention leading to faster progression of CKD [7]. Low birth weight has also been attributed to the increased risk of CKD [8].

Thyroid hormones are important for the development of kidneys and the maintenance of the internal environment of the body [9]. The thyroid hormones and kidney function are interrelated with each other [10]. The kidney not only participates in thyroid hormone metabolism and elimination, but it also serves as an important site for thyroid hormone action. Thyroid hormone secretion is disturbed in CKD patients as the hypothalamic pituitary thyroid axis gets influenced [11]. Alternatively, thyroid disturbance leads to altered kidney function by affecting water and electrolyte balance, GFR, kidney architecture, renal blood flow, and tubular function [12].

Low triiodothyronine (T3) is the commonest laboratory finding in CKD patients. Factors like metabolic acidosis and protein loss in uremic patients affect the functioning of the iodothyronine deiodinase enzyme leading to reduced conversion of thyroxine (T4) to T3 [13]. Inflammatory cytokines like tumor necrosis factor (TNF)-α and interleukin (IL)-1 get accumulated due to decreased clearance inhibiting the expression of 1 5’-deiodinase leading to low T3 [14]. Thyroid-stimulating hormone (TSH) levels may be normal or elevated in CKD patients but usually, there is a reduced response of pituitary receptors to thyrotropin-releasing hormone (TRH). They have both altered TSH circadian rhythm and glycosylation. Moreover, there is reduced clearance of TSH leading to increased half-life so the response of TSH to TRH gets blunted [13]. In CKD patients, T4 levels may be normal or reduced because of monodeiodinase action in the inner benzene ring leading to the formation of reverse T3, which passes from the vascular space to extravascular and intracellular spaces [13]. Hyperthyroidism is usually not associated with CKD in fact it may enhance CKD [15].

In view of the interrelationship between kidney function and thyroid hormone status and their variability, it is important for clinicians to understand their correlation. Despite a large number of available studies, there is a paucity of Indian data, especially in the northeastern region of India regarding their correlation. So, the present study was planned on CKD patients either on conservative management or hemodialysis to determine their thyroid profile status. To the best of our knowledge, no such study has been conducted in the Barak Valley region of the northeastern state of Assam. The outcome of our study may help to understand the comorbid conditions associated with CKD.

## Materials and methods

The present hospital-based, cross-sectional, observational study was conducted in the Department of Biochemistry, Silchar Medical College & Hospital, Assam, a tertiary care teaching hospital in Northeast India from September 2016 to August 2017 after being approved by the Institutional Ethics Committee (Silchar Medical College & Hospital; approval number: 10/06/2016).

Study participants and selection

The study comprised 50 patients of CKD between 40 and 70 years of age of both sexes with no previous history of any thyroid dysfunction, while 50 apparently healthy age and sex-matched individuals from the same ethnic population with normal renal function and no past history of thyroid disorders served as a control group. There were 35 males (70%) and 15 females (30%) in each group. Patients were recruited from both inpatient and outpatient departments of the medicine department and those attending the dialysis unit of the hospital. CKD patients with serum creatinine >5.5 mg/dl and urea level >55 mg/dl and a dipstick test positive for protein were included in the study based on their history, clinical signs, and symptoms. eGFR was calculated using a modification of diet in renal disease (MDRD) 4 variable formula [16]. All the cases were in stage 5 of CKD with eGFR < 15 ml/min/m^2^, of which 24 (48%) cases were on conservative management, while 26 (52%) were on hemodialysis. The study subjects participated voluntarily after they were explained the nature and purpose of the study. The written informed consent was obtained from the participants and their confidentiality was maintained.

Exclusion criteria included patients less than 40 years of age, family history of goiter or thyroid dysfunction, and those receiving concurrent treatment for thyroid disease and drugs known to affect thyroid hormone indices like glucocorticoids, salicylates, heparin, lithium, amiodarone, sulphonylurea, or phenobarbitone. All the participants included in the study underwent estimation for serum total triiodothyronine (TT3), total thyroxine (TT4), TSH, blood urea, and serum creatinine.

Sample collection and techniques

Taking all aseptic and antiseptic precautions, 2 ml of venous blood from the median cubital vein was collected after overnight fasting in a clot activator vial for TT3, TT4, TSH, blood urea, and serum creatinine estimation. The sample was left for 30 to 45 minutes and allowed to clot completely. The vial was then centrifuged at 3000 rpm for five minutes in a centrifuge machine. Precautions were taken to prevent hemolysis of the sample. The supernatant serum was used for the investigations or transferred to a clean, dry vial for storage if the estimations were not done in the same sitting. All chemicals used in the study were of analytical grade, and deionized water (Millipore) was used for various purposes.

The urease method and the Jaffe alkaline picrate method were used to calculate blood urea and serum creatinine on the Beckman Coulter Au 480 autoanalyzer (Beckman Coulter, Brea, CA). Estimation of TT3, TT4, and TSH was done in an autoanalyzer using the Access 2 Immunoassay System by Beckman Coulter using a chemiluminescent immunoassay. After biochemical estimations, the results obtained were statistically analyzed and compared between different groups of studies.

Statistical analysis

The data collected were entered into Microsoft Excel (Microsoft Corporation, Redmond, WA) and analyzed using GraphPad InStat version 3.00 for Windows (GraphPad Software, San Diego, CA). The data were expressed as mean, standard deviation (SD), standard error of the mean (SEM), and confidence interval (CI). The significance between different variables was tested using Student’s t-test. The chi-square test was used to analyze discrete variables. A two-tailed p-value of less than 0.05 was considered statistically significant.

## Results

The study subjects comprised 35 males (70%) and 15 females (30%) in each of the case and control groups. The mean age of the case and control groups was 55.32 ± 9.62 years and 54.48 ± 9.63 years, respectively. The maximum number of subjects in each of the two groups was in the age group of 54-60 years. All the subjects were investigated for blood urea, serum creatinine, TT3, TT4, and TSH. The eGFR was calculated using the 4-variable MDRD formula. All the cases were in stage 5 of CKD (eGFR < 15 ml/min/m^2^), out of which 36 (72%) had eGFR ≤ 10 ml/min/m^2^.

The mean blood urea level was 153.94 ± 72.50 (mg/dl) in cases and 22.91 ± 7.36 (mg/dl) in controls, while the mean serum creatinine level was 8.76 ± 3.48 (mg/dl) in cases and 0.75 ± 0.31 (mg/dl) in controls. In the unpaired t-test between the case and control groups, the two-tailed p-value was <0.0001 for both blood urea and serum creatinine (Table 1).

**Table 1:** Mean value of blood urea and serum creatinine in the studied groups. SD: standard deviation; SEM: standard error of the mean; CI: confidence interval.

Variables	Blood urea (mg/dl)		Serum creatinine (mg/dl)	
	Cases	Control	Cases	Control
Mean	153.94	22.91	8.76	0.75
SD	72.50	7.36	3.48	0.31
SEM	10.25	1.04	0.49	0.04
95% CI	133.32-174.57	20.81-25.06	7.78-9.76	0.66-0.83
P-value	<0.0001		<0.0001	

TT3 level was reduced in all 50 cases. TT4 level was normal in 31 (62%), reduced in 18 (36%), and high in one (2%) case. TSH level was high in 38 (76%), reduced in one (2%), and normal in 11 (22%) cases (Table 2).

**Table 2:** Analysis of TT3, TT4, and TSH among CKD patients. TT3: total triiodothyronine; TT4: total thyroxine; TSH; thyroid-stimulating hormone; CKD: chronic kidney disease.

	No. (% age) of CKD patients		
Variables (normal range)	Normal	Low	High
TT3 (81-171 ng/dl)	-	50 (100%)	-
TT4 (4-12 µg/dl)	31 (62%)	18 (36%)	1 (2%)
TSH (0.4-4.0 µIU/ml)	11 (22%)	1 (2%)	38 (76%)

The mean blood level of TT3, TT4, and TSH in cases was 40.77 ± 12.58 (ng/dl), 6.61 ± 3.06 (µg/dl), and 16.92 ± 26.97 (µIU/ml), respectively, while in control, it was 109.27 ± 22.26 (ng/dl), 8.94 ± 1.91 (µg/dl), and 2.29 ± 1.24 (µIU/ml), respectively. The unpaired t-tests between the case and control groups in both TT3 and TT4 were extremely significant with a two-tailed p-value <0.0001, while for TSH, it was significant with a two-tailed p-value of 0.0002 (Table 3).

**Table 3:** Mean value of TT3, TT4, and TSH in the studied groups. TT3: total triiodothyronine; TT4: total thyroxine; TSH; thyroid-stimulating hormone; SD: standard deviation; SEM: standard error of the mean; CI: confidence interval.

	TT3 (ng/dl)		TT4 (µg/dl)		TSH (µIU/ml)	
	Cases	Control	Cases	Control	Cases	Control
Mean	40.77	109.27	6.61	8.94	16.92	2.29
SD	12.58	22.26	3.06	1.91	26.97	1.24
SEM	1.77	3.14	0.43	0.27	3.81	0.17
95% CI	37.20-44.35	102.94-115.60	5.74-7.48	8.39-9.48	9.25-24.60	1.93-2.64
P-value	<0.0001		<0.0001		0.0002	

The mean levels of TT3, TT4, and TSH in cases on conservative treatment were 46.76 ± 11.84 (ng/dl), 8.10 ± 2.381 (µg/dl), and 6.19 ± 4.07 (µIU/ml), respectively, while in cases on hemodialysis, the mean levels were 34.48 ± 11.21 (ng/dl), 5.24 ± 3.019 (µg/dl), and 26.85 ± 34.59 (µIU/ml), respectively. In the unpaired t-test between cases on conservative treatment and cases on hemodialysis, the two-tailed p-value was 0.0005, 0.0006, and 0.0055, respectively (Table 4).

**Table 4:** Analysis of thyroid hormone in cases on the basis of treatment modalities (conservative or hemodialysis). TT3: total triiodothyronine; TT4: total thyroxine; TSH; thyroid-stimulating hormone; SD: standard deviation; SEM: standard error of the mean; CI: confidence interval.

	TT3 (ng/dl)		TT4 (µg/dl)		TSH (µIU/ml)	
	Conservative	Hemodialysis	Conservative	Hemodialysis	Conservative	Hemodialysis
Mean	46.76	34.48	8.10	5.24	6.19	26.85
SD	11.84	11.21	2.381	3.019	4.07	34.59
SEM	2.49	2.24	0.4859	0.5921	0.8316	6.785
95% CI	42.02-51.49	30.17-38.78	7.102-9.112	4.025-6.465	4.449-7.890	12.881-40.834
P-value	0.0005		0.0006		0.0055	

## Discussion

The kidney contributes to the clearance of iodine principally through glomerular filtration. Reduced GFR in CKD patients leads to a decrease in iodine clearance. This causes an increase in plasma iodide concentration and a rise in thyroidal tissue iodide uptake. An increase in total body inorganic iodide blocks thyroid hormone production by inhibiting the pituitary thyroid axis (Wolff-Chaikoff effect). These changes may describe the increased frequency of hypothyroidism in CKD patients [17].

The present study was conducted on 50 CKD patients and compared with healthy controls for their thyroid hormone status. The most common thyroid function abnormality in the current study was low TT3 found in all the CKD patients and their mean TT3 was significantly reduced compared to healthy control. The majority of CKD patients in the present study showed normal or reduced TT4 levels; however, the mean TT4 showed a statistically significant reduction compared to the healthy controls. The TSH level in the present study was found to be mostly elevated or normal while the mean TSH was significantly increased in CKD patients compared to control. Similar results were obtained by Falhi et al. who investigated 50 CKD patients aged 20 to 50 years and showed a highly significant decrease (P < 0.01) in T3 and T4 levels and an increase in TSH level as compared with controls [18]. While the study by Khatiwada et al. observed a non-significant decrease in T3 and T4 levels but a significant rise in the TSH level was observed in CKD patients [19]. Rajagopalan et al. in 2013 found a significant decrease in T3 and T4 with unchanged TSH in CKD patients as compared to controls [20]. A previous study done in Iraq on CKD patients receiving either conservative management or regular hemodialysis showed a significant reduction of TT3 and TT4; however, the level of TSH did not show significant alterations compared to the control group [21]. In 2019, Alshammari et al. discovered a non-significant correlation between the incidence of hypothyroidism and a decrease in GFR, whereas Toda et al. discovered a statistically significant correlation [22,23].

Analysis of mean TT3, TT4, and TSH was also done between CKD patients receiving conservative management and those on hemodialysis. The CKD patients on hemodialysis showed a significant reduction in mean TT3 and TT4, while mean TSH showed a significant increase than those on conservative treatment. Most CKD patients on hemodialysis are euthyroid; however, hypothyroidism is not uncommon, which is usually associated with a reduction in serum total and free T3. This decline is associated with endothelial damage, systemic acidosis, and inflammation [24]. Malik revealed no significant difference in TT3 and TT4 between the patients on conservative management and those on regular hemodialysis [21]. The study by Kayima et al. revealed that the mean values of TT4, TT3, free T4, and free T3 were significantly low, while the mean TSH level was significantly higher in patients compared with controls (P < 0.01), while no significant differences were found in all the parameters between those on hemodialysis and conservative management (P ˃ 0.05) [25].

Limitations of the study

The CKD patients included in the study were not categorized based on their etiology, so the individual causal relationship between CKD and their thyroid dysfunction could not be assessed. The iodine status of the participants was not assessed, which may be a limitation, as excess iodine nutrition or iodine deficiency may contribute to thyroid disorders. The current study was not strict about the frequency and duration of hemodialysis while selecting the patients, which may affect the thyroid hormone status.

## Conclusions

The present cross-sectional, hospital-based observational study observed a significant reduction in mean TT3 and TT4 along with elevation of mean TSH in uremic patients irrespective of the mode of treatment and duration of disease as compared to healthy controls. In light of the results revealed from the present and most of the previous reports, there is a likelihood of dysfunction in the activities of thyroid hormones with a decline in renal function. Early identification of thyroid dysfunction and its intricate relationship with kidney function may be important in planning treatment strategies. Clinicians must take into account thyroid disorders along with treating renal abnormality in CKD patients.
